# Is the Hospital Anxiety and Depression Scale (HADS) a valid measure in a general population 65–80 years old? A psychometric evaluation study

**DOI:** 10.1186/s12955-017-0759-9

**Published:** 2017-10-04

**Authors:** Ingrid Djukanovic, Jörg Carlsson, Kristofer Årestedt

**Affiliations:** 1 Faculty of Health and Life Sciences, Linnaeus University, 391 82 Kalmar, SE Sweden; 2 Department of Research, Kalmar County Hospital, Kalmar, Sweden; 3 Department of Medical and Health Sciences, Linköping University, Linköping, Sweden

**Keywords:** Validation, Psychometric, Elderly, Anxiety, Depression

## Abstract

**Background:**

The HADS (Hospital Anxiety and Depression Scale) aims to measure symptoms of anxiety (HADS Anxiety) and depression (HADS Depression). The HADS is widely used but has shown ambiguous results both regarding the factor structure and sex differences in the prevalence of depressive symptoms. There is also a lack of psychometric evaluations of the HADS in non-clinical samples of older people. The aim of the study was to evaluate the factor structure of the HADS in a general population 65–80 years old and to exam possible presence of differential item functioning (DIF) with respect to sex.

**Methods:**

This study was based on data from a Swedish sample, randomized from the total population in the age group 65–80 years (*n* = 6659). Confirmatory factor analyses (CFA) were performed to examine the factor structure. Ordinal regression analyses were conducted to detect DIF for sex. Reliability was examined by both ordinal as well as traditional Cronbach’s alpha.

**Results:**

The CFA showed a two-factor model with cross-loadings for two items (7 and 8) had excellent model fit. Internal consistency was good in both subscales, measured with ordinal and traditional alpha. Floor effects were presented for all items. No indication for meaningful DIF regarding sex was found for any of the subscales.

**Conclusions:**

HADS Anxiety and HADS Depression are unidimensional measures with acceptable internal consistency and are invariant with regard to sex. Despite pronounced ceiling effects and cross-loadings for item 7 and 8, the hypothesized two-factor model of HADS can be recommended to assess psychological distress among a general population 65–80 years old.

## Background

Psychological distress in terms of depression and anxiety is a growing problem among older people, with a prevalence of depression in European countries at 12% for people aged 65 years or over [1]. For anxiety the corresponding prevalence varies from 1% to 14% in North America and Europe [2]. Late-life depression can have serious consequences such as increased comorbidity with physical illness, reduced function and increased risk of suicide [3]. Also anxiety in this age group can lead to considerable distress and functional impairment [4]. As there are a considerable number of older people who suffer from symptoms of anxiety and/or depression, there is a need for a brief and feasible instrument to identify people at risk but also for evaluation of interventions and for research.

The Hospital Anxiety and Depression Scale (HADS) is a frequently used self-rating scale developed to assess psychological distress in non-psychiatric patients. It consists of two subscales, Anxiety and Depression [5]. Overall, it has demonstrated satisfactory psychometric properties in different groups; in primary care patients [6], cognitively intact nursing home patients [7], cancer inpatients [8] and in general populations [6, 9]. However, previous studies have suggested different factor structures of the HADS. The hypothesized two-factor structure is most often confirmed [8, 10, 11] but studies have also suggested one factor [12], three factors [13] and also four factors [14]. In addition, few studies have evaluated the factor structure in older populations specifically, and existing studies show divergent factor structures as well. A study by Helvik et al. [15] supported a two-factor model in a sample of hospitalized patients 65 years and older. However, three items in their study did not load on the expected factor suggested by the constructors [5]. One study in a community-dwelling population aged 60–80 years confirmed the two-factor structure as the most plausible and also more clinical relevant in comparison with a three-factor model [16]. Also Gale et al. [10] found a two-factor model as more appropriate compared to a three factor model in non-clinical populations of older men and women. In contrast, a three-factor model was suggested in a study regarding older veterans (> 65 years) with limb amputation [13]. As knowledge about the latent structure of the HADS in an older population is limited and has shown contradictory results, there is a need to further evaluate the factor structure in a general population of older people.

Previous research has commonly shown that psychological distress is more prevalent among women than men [2, 17]. However, some studies have shown no statistically significant differences between women and men regarding prevalence of depression [15, 18] whereas Martin et al. [19] showed a higher prevalence for men. A possible explanation to these diverging results could be heterogeneity regarding study design, population and measurements. As previous research also has shown ambiguous results when using the HADS [20, 21], there is a need to examine if HADS is an invariant measure for psychological distress for women and men.

Differential item functioning (DIF) is an often overlooked aspect of validity and occurs when different subgroups respond differently to specific items within a scale, after matching on the underlying latent construct that the item is intended to measure [22]. If DIF is presented it implies that the scale is not measuring the same thing for all respondents and thus might lead to incorrect conclusions.

The aim of the study was to evaluate the factor structure of the Hospital Anxiety and Depression scale (HADS) in a general population 65–80 years old and further to exam the possible presence of differential item functioning (DIF) with respect to sex.

## Method

This validation study was based on a cross-sectional survey including a random sample of individuals (*n* = 9968) selected from the total Swedish population aged 65–80 years (*N* = 1.276.307). The main aim was to investigate the prevalence of, and association between depressive symptoms and loneliness in relation to age and sex [21]. Ethical approval was obtained from the Regional Ethic Review Board in Stockholm, Sweden (No. 2010/823–314/4).

### Sample and procedures

Participants were randomly selected from a national register of the total population, which includes all persons registered as residents in Sweden. The inclusion criterion was being in the age group 65–80 years. The study was based on postal questionnaires. Statistics Sweden performed the randomization and distributed the questionnaires together with information about the study emphasizing voluntariness to participate and anonymity in relation to researchers. Questionnaires and follow-up letters were sent to non-respondents after three weeks and resulted in a response rate of 67.0% for the total sample (66.6% for women and 67.1% for men). For this psychometric evaluation study, 37 questionnaires had missing data in all items regarding HADS and were therefore excluded, leaving a final sample of 6622 participants.

### The questionnaire

The questionnaire was divided into two parts; one part was specifically reflecting e.g. demographics, morbidity, and pharmacological treatment while the other part concerned psychological distress, symptoms of anxiety and depression, measured with HADS.

The HADS aims to measure symptoms of anxiety and depression and consists of 14 items, seven items for the anxiety subscale (HADS Anxiety) and seven for the depression subscale (HADS Depression). HADS Anxiety focus mainly on symptoms of generalized anxiety disorder and HADS Depression is focused on anhedonia, the main symptom of depression [23]. Each item is scored on a response-scale with four alternatives ranging between 0 and 3. After adjusting for six items that are reversed scored, all responses are summed to obtain the two subscales. Recommended cut-off scores according to Zigmond & Snaith [5] are 8–10 for doubtful cases and ≥11 for definite cases. An optimal balance between sensitivity and specificity was found using a cut-off score of 8 or above for both HADS Anxiety and HADS Depression [6].

### Data analysis

Demographic characteristics are presented with descriptive statistics (frequencies, means, standard deviations, medians and interquartile ranges) and differences between sexes were analyzed with independent sample t-test and chi-square test.

An item analysis was conducted to evaluate score distributions, floor/ceiling effects, and missing data patterns. Analyses of distribution were based on descriptive statistics for ordinal data. However, mean and standard deviations were also calculated for comparisons with previous studies. The D’Agostino test was conducted to evaluate if item and scale scores deviated significantly from a normal distribution. Floor and ceiling effects, which refer to the proportions of participants with the lowest (floor) and highest (ceiling) possible scores, were evaluated using frequency distributions. Up to 20% floor/ceiling effects were considered acceptable in the present study. To test if the data were completely missing at random (MCAR), Little’s chi-squared test for MCAR was conducted for each scale separately. Homogeneity was evaluated with inter-item correlations based on polychoric correlations.

A confirmatory factor analysis (CFA) was conducted to evaluate the hypothesized two-factor structure of the HADS (model I); 7 item measuring anxiety and 7 item measuring depression, without any other modifications. As the model did not perfectly fit the data, a second model was evaluated (model II); two-factors with cross-loadings for item 7 and 8. As the items were highly skewed distributed with pronounced floor effects, a third model was evaluated to identify which impact this problem had on the factor structure (model III); a two-factor model with cross-loadings for item 7 and 8 together with collapsed response categories (category 2 and 3 for all items). The items were treated as ordered indicator variables and consequently a diagonally weighted least square method (WLSMV), based on a polychoric correlation matrix, was used to estimate the parameters of the models. Different goodness-of-fit statistics were used to evaluate the CFA models. A non-significant chi-square test indicates a perfect model fit between model and data. However, since this test is highly sensitive for large sample sizes it should be interpreted with caution. Therefore we used the following goodness-of-fit criteria; root mean square error of approximation (RMSEA) ≤ 0.06, comparative fit index (CFI) ≥ 0.95 and Tucker-Lewis index (TLI) ≥ 0.95 [24].

To evaluate internal consistency reliability, an ordinal variant of Cronbach’s alpha was calculated [25]. This calculation is based on polychoric correlations rather than Pearson correlations, but is interpreted in the same way as the traditional Cronbach’s alpha. Thus, alpha values above 0.7 indicate sufficient internal consistency reliability [26] For comparisons, also traditional Cronbach’s alpha was calculated.

Examination of differential item functioning (DIF) for sex was conducted for each item using ordinal regression analyses. This method enables to test for both uniform (effects of group differences) and non-uniform DIF (effects of differences in group ability) [22, 27]. In the first step (Block I), the item responses were treated as outcome variables predicted by the conditional variable (i.e. total score for HADS Anxiety and HADS Depression respectively). In the second step (Block II), the grouping variable (i.e. sex) was added as covariate to detect uniform DIF. In the third step (Block III), the interaction term between the conditional variable and group variable (i.e., sex × HADS Anxiety and sex × HADS Depression) were added as covariates to test for non-uniform DIF [22]. The change in McFadden R^2^ between the three models was used to evaluate the effect size of DIF. For an item to be classified as showing DIF, the two degree of freedom chi-squared test in logistic regression must have a *p*-value <0.01 and the effect size measure have to be at least R^2^ ≥ 0.13 [22].

The analyses were conducted with the SPSS Statistics 20.0 (IBM Corp, Armonk, NY, USA), Mplus 7.4 (Muthén & Muthén, Los Angeles, CA, USA) and R 3.3.0 software (the R Foundation for Statistical Computing, Vienna Austria).

## Results

### Sample characteristics

The overall mean age was 71.2 years (SD = 4.5). The sample consisted of almost as many men as women, 48.4% and 51.6% respectively. A majority were married/cohabitating (70.5%), was retired (80.2%) and reported primary school as the highest education level (49.1%). The proportion of participants scoring HADS Anxiety were 10.7% for the entire sample, significantly more common among women than men (14.1% vs. 7.0%, *p* < 0.001). Corresponding results for HADS Depression ≥8 was 9.8%, and in opposite to anxiety, significantly more common for men than women (10.6% vs. 9.1%, *p* < 0.05). Antidepressant medication was prescribed for 8.0% and anxiolytic medication had nearly the same prescription rate (7.0%). Less than 1% had visited psychologist or welfare officer during the last three months (Table 1).

**Table 1:** Demographic characteristics of the study population in relation to sex

	Total (n = 6659)	Women (*n* = 3436)	Men (*n* = 3223)	*p*-value
Age, M (SD)	71.2 (4.5)	71.3 (4.5)	71.1 (4.5)	0.328^a^
Married/Cohabiting, n (%)	4611 (70.5)	2107 (62.4)	2504 (79.0)	< 0.001^b^
Education, n (%)				< 0.001^b^
Incomplete primary school	405 (6.1)	196 (5.9)	209 (6.6)	
Primary school	3189 (47.9)	1723 (51.4)	1466 (46.5)	
High School/College	1662 (24.9)	765 (22.8)	897 (28.5)	
University	1243 (18.7)	665 (19.9)	578 (18.4)	
Still Working, n (%)	579 (9.2)	224 (6.9)	355 (11.6)	< 0.001^b^
Morbidity, n (%)				
Diabetes	845 (16.4)	354 (13.6)	491 (19.2)	< 0.001^b^
Asthma	558 (11.2)	327 (12.8)	231 (9.5)	0.001^b^
Hypertension	2924 (48.3)	1520 (46.6)	1476 (50.0)	< 0.001^b^
Pharmacological treatment, n (%)				
Antidepressant medication	392 (8.0)	269 (10.8)	123 (5.1)	< 0.001^b^
Anxiolytic medication	340 (7.0)	222 (4.6)	118 (2.4)	< 0.001^b^
Sleeping pills	946 (18.8)	659 (25.2)	287 (11.8)	< 0.001^b^
Visits last three months, n (%)				
Psychologist	39 (0.8)	28 (1.1)	11 (0.4)	0.036^b^
Welfare officer	55 (0.6)	39 (1.4)	16 (0.6)	0.014^b^
HADS score, Mdn (q1-q3)				
Anxiety	2 (1–5)	3 (1–6)	2 (0–4)	< 0.001^c^
Depression	3 (1–5)	2 (1–5)	3 (1–5)	< 0.05^c^
HADS score ≥ 8, n (%)				
Anxiety	697 (10.7)	476 (14.1)	221 (7.0)	< 0.001^b^
Depression	641 (9.8)	307 (9.1)	334 (10.6)	< 0.05^b^

*HADS* Hospital Anxiety and Depression Scale

^a^Independent sample t-test

^b^Pearson chi-square test

^c^Mann-Whitney U test

### Item score statistics

Item scores for both HADS Anxiety and HADS Depression deviated significantly from a normal distribution, graphically (normal probability plot) and statistically (D’Agostino test (*p* < 0.001). No ceiling effects were presented, but all items showed floor effects. The score distribution for the lowest response alternative ranged between 52.8% and 77.7% for the items in HADS Anxiety and between 36.1% and 76.4% for HADS Depression (Table 2).

**Table 2:** Item and scale score statistics (*n* = 6622)

			Score distribution, %				
	Mdn (q1-q3)	M (SD)	0	1	2	3	Missing data
HADS Anxiety	2 (1–5)	3.23 (3.23)					
1. I feel tense and wound up	0 (0–1)	0.53 (0.63)	**52.8**	41.3	4.1	1.0	0.8
3. I get sort of frightened as if something awful is about to happen	0 (0–1)	0.34 (0.59)	**70.7**	25.3	3.0	1.0	0.8
5. Worrying thoughts go through my mind	0 (0–1)	0.49 (0.71)	**60.3**	30.6	5.9	2.2	1.4
7. I can sit at ease and feel relaxed	1 (0–1)	0.59 (0.64)	**47.9**	44.7	6.4	0.7	0.2
9. I get sort of frightened feeling like “butterflies in the stomach”	0 (0–1)	0.42 (0.58)	**62.0**	33.8	3.0	0.5	0.6
11. I feel restless as if I have to be on the move	0 (0–1)	0.60 (0.73)	**53.1**	33.7	11.6	1.1	0.6
13. I get sudden feelings of panic	0 (0–0)	0.25 (0.52)	**77.7**	18.8	2.4	0.6	0.6
HADS Depression	3 (1–5)	3.26 (3.04)					
2. I still enjoy the things I used to enjoy	0 (0–1)	0.45 (0.59)	**58.7**	37.0	2.7	0.7	0.9
4. I can laugh and see the funny side of things	0 (0–1)	0.31 (0.57)	**72.6**	22.7	3.3	0.6	0.7
6. I feel cheerful	0 (0–1)	0.34 (0.60)	**72.2**	21.3	5.3	0.6	0.7
8. I feel as if I have slowed down	1 (0–1)	0.76 (0.69)	**36.1**	53.4	7.7	2.2	0.6
10. I have lost interest in my appearance	0 (0–1)	0.57 (0.72)	**54.4**	34.0	9.6	1.2	0.8
12. I look forward with enjoy to things	0 (0–1)	0.53 (0.70)	**56.8**	33.2	8.0	1.2	0.7
14. I can enjoy a good book, or radio or TV program	0 (0–0)	0.29 (0.59)	**76.4**	19.0	2.7	1.5	0.4

Floor and/or ceiling effects are marked in bold, defined if more than 20% of the participants used the lowest and/or highest possible scores

*HADS* Hospital Anxiety and Depression Scale

The presence of missing data was low and ranged between 0.6% and 1.4% for items in HADS Anxiety and 0.2% and 0.9% for items in HADS Depression (Table 2). However, according to the Little MCAR test data was not complete missing at random for either HADS Anxiety (χ^2^(120) = 278.6, *p* < 0.001) or HADS Depression (χ^2^(149) = 278.6, p < 0.001).

The homogeneity was satisfactory, the mean inter-item correlations were 0.61 (SD = 0.07, range = 0.51–0.75) for the HADS Anxiety and 0.51 (SD = 0.10, range = 0.35–0.68) for HADS Depression (Table 3).

**Table 3:** Inter-item correlation matrix based on polychoric correlations, pairwise deletion (n = 6622)

Items	1	2	3	4	5	6	7	8	9	10	11	12	13	14
1	1.000													
2	0.398	1.000												
3	0.586	0.374	1.000											
4	0.469	0.575	0.501	1.000										
5	0.640	0.405	0.752	0.542	1.000									
6	0.489	0.514	0.495	0.659	0.576	1.000								
7	0.588	0.445	0.511	0.557	0.561	0.568	1.000							
8	0.572	0.474	0.542	0.544	0.582	0.616	0.536	1.000						
9	0.628	0.377	0.699	0.495	0.676	0.470	0.529	0.552	1.000					
10	0.290	0.404	0.300	0.396	0.304	0.473	0.344	0.436	0.270	1.000				
11	0.641	0.351	0.518	0.443	0.551	0.482	0.581	0.527	0.559	0.330	1.000			
12	0.462	0.640	0.460	0.672	0.507	0.680	0.523	0.587	0.446	0.504	0.442	1.000		
13	0.617	0.368	0.684	0.484	0.674	0.510	0.556	0.572	0.719	0.358	0.594	0.458	1.000	
14	0.293	0.356	0.284	0.437	0.288	0.477	0.446	0.367	0.254	0.351	0.356	0.446	0.346	1.000

### Factor structure

**Fig. 1:**
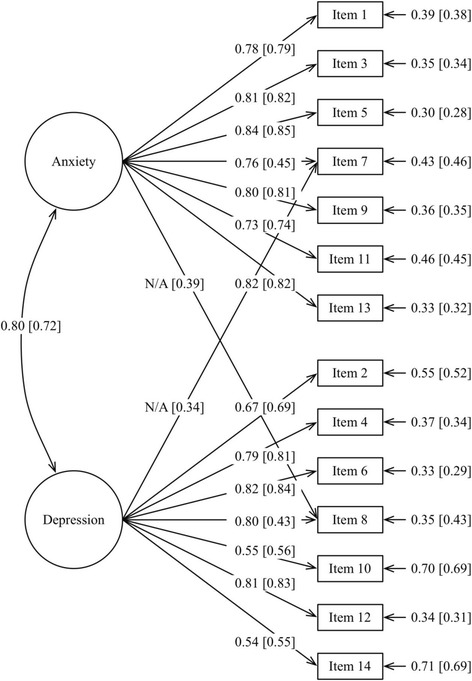
Parameter estimates (i.e., factor correlations, factor loadings, cross-loadings and residual variances) from model I (outside brackets) and model II (inside brackets)

The two-factor model (I) without modifications showed a reasonable but not perfect fit between model and data. The factor loadings ranged between 0.73 and 0.84 for HADS Anxiety and between 0.54 and 0.82 for HADS Depression. The RMSEA was close but still above the critical value of ≤0.06 (RMSEA = 0.07). In contrast, both CFI and TLI indicated a good model fit according to the critical value of ≥0.95 (CFI = 0.97, TLI = 0.96). Based on the modification index and recent research [15, 28], item 7 and 8 were allowed to cross-load on both factors (i.e., HADS Anxiety and HADS Depression) in model II. This model demonstrated an excellent fit according to all goodness-of-fit indices (RMSEA = 0.05, CFI = 0.98, TLI = 0.98) and no further need for revision according to the modification index. However, factor loadings as well as cross-loadings for item 7 and 8 decreased <0.5 for both HADS Anxiety and HADS Depression. Furthermore, the residual variance increased for both items compared with model I. All other factor loadings were >0.5 and ranged between 0.74 and 0.85 for HADS Anxiety and 0.55 and 0.84 for HADS Depression (Table 4, Fig. 1).

**Table 4:** Goodness-of-fit indices for the confirmatory factor analyses models (n = 6622)

	χ^2^ goodness-of-fit			RMSEA				
Models	χ^2^	df	*p*-value	RMSEA	90% CI	*p*-value	CFI	TLI
I Baseline	2405.2	76	< 0.001	0.068	0.066–0.070	< 0.001	0.965	0.958
II Cross-loadings	1280.3	74	< 0.001	0.050	0.047–0.052	0.599	0.982	0.978
III Cross-loadings and collapsed response categories	1126.4	73	< 0.001	0.047	0.044–0.049	0.988	0.984	0.980

Model I = baseline model without modifications; Model II = model with cross-loadings for item 7 and 8; Model III = model with cross-loadings for item 7 and 8 and collapsed response categories (category 2 and 3)

Goodness-of-fit indices for excellent model fit: *RMSEA* root mean square error of approximation (≤ 0.06), *CFI* comparative fit index (≥ 0.95), *TLI* Tucker-Lewis index (≥ 0.95)

As the item scores were highly skewed distributed, a third model was evaluated to address this problem, in which category 2 and 3 were collapsed. This third model demonstrated model fit at the same level as model II (RMSEA = 0.05, CFI = 0.98, TLI = 0.98) (Table 4). In this model, factor loadings varied between 0.33 and 0.83 for HADS Anxiety and 0.40 and 0.84 for HADS Depression. As in model II, only factor loadings and cross-loadings for item 7 and 8 were < 0.5.

### Internal consistency reliability

The internal consistency reliability, assessed with ordinal alpha, was 0.92 for HADS Anxiety and 0.88 for HADS Depression. The corresponding internal consistency measured with traditional Cronbach’s alpha was 0.87 and 0.81 respectively.

### Differential item functioning

The results from the ordinal regressions analysis are presented in Tables 5 and 6. The conditional variable (i.e. HADS scale scores) was significantly associated with all item responses for both HADS Anxiety and HADS Depression in Block I. The group variable (i.e. sex) was also significantly associated with all items in both HADS Anxiety and HADS Depression (*p* < 0.001). The same findings were demonstrated when the interaction term (i.e. sex x HADS scale scores) were included in block III. However, the explained variance according to the McFadden pseudo R^2^ did not increase more than up to 0.01 for the items in HADS Anxiety and 0.02 for HADS Depression across the three models (Block I-III). Based on this, no indication of meaningful DIF for sex was detected.

**Table 5:** Detection of uniform and non-uniform differential item functioning for sex in Hospital Anxiety and Depression Scale - Anxiety (HADS-A), based on ordinal regression

		Block I			Block II			Block III			
Items	Predictors	OR	p-value	R^2^	OR	p-value	R^2^	OR	p-value	R^2^	Model comparisons^a^
1	HADS-A	2.17	< 0.001	0.412	2.17	< 0.001	0.414	2.53	< 0.001	0.415	A
	Sex (male)				1.30	< 0.001		0.94	< 0.001		B
	Sex x HADS-A							0.91	< 0.001		C
3	HADS-A	2.01	< 0.001	0. 418	1.99	< 0.001	0.425	2.22	< 0.001	0. 425	A
	Sex (male)				0.56	< 0.001		0.41	< 0.001		B
	Sex x HADS-A							0.94	< 0.001		C
5	HADS-A	2.25	< 0.001	0.438	2.24	< 0.001	0.443	2.46	< 0.001	0.444	A
	Sex (male)				0.60	< 0.001		0.46	< 0.001		B
	Sex x HADS-A							0.94	< 0.001		C
7	HADS-A	1.90	< 0.001	.333	1.92	< 0.001	0.335	2.12	< 0.001	0.336	A
	Sex (male)				1.35	< 0.001		1.11	< 0.001		B
	Sex x HADS-A							0.94	< 0.001		C
9	HADS-A	2.14	< 0.001	.432	2.13	< 0.001	0.442	2.24	< 0.001	0.442	A
	Sex (male)				0.51	< 0.001		0.45	< 0.001		B
	Sex x HADS-A							0.97	< 0.001		C
11	HADS-A	1.90	< 0.001	0.334	1.96	< 0.001	0.342	2.16	< 0.001	0.343	A
	Sex (male)				1.84	< 0.001		1.47	< 0.001		B
	Sex x HADS-A							0.94	< 0.001		C
13	HADS-A	2.00	< 0.001	0.435	2.00	< 0.001	0.435	2.29	< 0.001	0.436	A
	Sex (male)				1.05	< 0.001		0.67	< 0.001		B
	Sex x HADS-A							0.92	< 0.001		C

^a^Model comparison is based on the χ^2^ difference test between the models, with Bonferroni corrected p-values (*p* < 0.017). Significant differences are reported as: A = Block I & II, B = Block I & III, C = Block II & III

**Table 6:** Detection of uniform and non-uniform differential item functioning for sex in Hospital Anxiety and Depression Scale - Depression (HADS-D), based on ordinal regression

		Block I			Block II			Block III			
Items	Predictors	OR	p-value	R^2^	OR	*p*-value	R^2^	OR	*p*-value	R^2^	Model comparisons^a^
2	HADS-D	1.83	< 0.001	0.313	1.84	< 0.001	0.313	1.92	< 0.001	0.313	A
	Sex (male)				0.88	< 0.001		0.79	< 0.001		–
	Sex x HADS-D							0.98	< 0.001		–
4	HADS-D	1.95	< 0.001	0.375	1.98	< 0.001	0.385	1.98	< 0.001	0.385	A
	Sex (male)				1.96	< 0.001		0.51	< 0.001		B
	Sex x HADS-D							1.00	< 0.001		–
6	HADS-D	2.10	< 0.001	0.411	2.11	< 0.001	0.411	1.93	< 0.001	0.412	–
	Sex (male)				0.88	< 0.001		1.18	< 0.001		–
	Sex x HADS-D							1.06	< 0.001		–
8	HADS-D	2.04	< 0.001	0.323	2.07	< 0.001	0.333	1.94	< 0.001	0.333	A
	Sex (male)				0.54	< 0.001		0.62	< 0.001		B
	Sex x HADS-D							1.05	< 0.001		–
10	HADS-D	1.73	< 0.001	0.238	1.70	< 0.001	0.248	1.68	< 0.001	0.248	A
	Sex (male)				1.82	< 0.001		1.87	< 0.001		B
	Sex x HADS-D							1.01	< 0.001		–
12	HADS-D	2.44	< 0.001	0.441	2.44	< 0.001	.441	2.32	< 0.001	0.441	–
	Sex (male)				0.85	< 0.001		0.98	< 0.001		–
	Sex x HADS-D							1.03	< 0.001		–
14	HADS-D	1.56	< 0.001	0.216	1.57	< 0.001	0.238	1.55	< 0.001	0.238	A
	Sex (male)				2.52	< 0.001		2.67	< 0.001		B
	Sex x HADS-D							1.01	< 0.001		–

^a^Model comparison is based on the χ^2^ difference test between the models, with Bonferroni corrected p-values (p < 0.017). Significant differences are reported as: A = Block I & II, B = Block I & III, C = Block II & III

## Discussion

In the present study, the psychometric properties of the HADS have been evaluated in a large general population of older people. Overall, the HADS showed to be a valid instrument to measure psychological distress in the current population. The original two-factor structure was confirmed, internal consistency was satisfactory and no DIF for sex was detected. Problems with floor effects were shown for all items.

The distribution of item responses was highly skewed towards lower scores and floor effects were shown for all items. However, all of the item response alternatives were endorsed which indicate that all response categories are relevant. A potential problem with this skewed distribution and floor/ceiling effects could be a negative impact on sensitivity and responsiveness [29]. This has been seen in other studies using the HADS [30, 31] and could therefore be expected. Further, this study was based on data from a general population where a limited proportion has shown to have symptoms of anxiety and depression. Thus, this problem is probably related to the sample rather than the instrument.

According to the Little MCAR test, missing data was not completely missing at random which indicate a systematic drop out. However, the number of missing responses was very low. Additionally, as many other statistical tests, the Little MCAR test is sensible to large sample sizes and a statistically significant result does not necessarily imply that it is clinically important [32]. The low rate of missing data indicates that the items are easy to understand and that the instrument is not too extensive and burdensome to complete for the respondents.

The CFA in the present study showed support for the hypothesized two-factor structure with two latent variables, anxiety and depression, which also is demonstrated in previous research regarding community-based healthy older people [10, 16]. However, our results identified problems with cross-loadings for item 7 and 8. This problem has been addressed in previous studies [15, 20]. After these items were allowed to cross-load on both factors (model II), the model fit was excellent according to all indices. It has been suggested that item 8 (“I feel like as if I have slowed down”) could be interpreted as age-related slowing down [15] and that item 7 (“I can sit at ease and feel relaxed”) both refers to psychomotor agitation and the anhedonia domain of the depression subscale and therefore loads both into the anxiety and depression factor [28]. This may explain why these two items seem to be indicators for both anxiety and depression. Even if the model fit increased in model II, the cross-loadings resulted in poor factor loadings below 0.5 and increased residual variances for both item 7 and 8. These findings indicate that the original two-factor model should be preferred despite that the RMSEA is above 0.06. Using the hypothesized two-factor model would also facilitate comparisons between studies. However, this problem needs to be addressed in further studies and users should be aware of this limitation of the HADS.

According to the skewed distribution with few responses on the third (2) and fourth (3) category, these two were collapsed in order to examine if this would increase the model fit further. This third model resulted in excellent fit, very close to the findings from model II. This finding indicates that the skewed distributions, with pronounced floor effects, did not have any serious effect on the factor structure. In addition, this third model was evaluated for statistical reasons and should not be applied for clinical use.

Although anxiety and depression are known to represent two different constructs, they are highly correlated. In our study the correlation between the two latent factors was strong, which is consistent with the understanding that there are symptomatic overlaps between anxiety and depression [33].

The internal consistency is well supported by both ordinal as well as traditional Cronbach’s alpha values for both HADS Anxiety and HADS Depression. This is similar to findings from studies in the same age group [2, 16] and thus supports the robustness of the scale for older people.

Our results show that the HADS can be used to make invariant comparisons between men and women even if the group variable and interaction term was significantly associated with item responses for all items. With a large sample, also small and meaningless associations will be highly significant. Therefore, pseudo R^2^ changes should be used to exam DIF rather than statistical significance. The effect measured with McFadden R^2^ was low which implies that no meaningful DIF was present. According to Zumbo, [22] R^2^ changes above 0.130 are required to determine the presence of DIF. This criteria has in later research been criticized for being too liberal [34] but even when more conservative criterion (R^2^ ≥ 0.035) suggested by Jodoin & Giel, [35] was applied, no meaningful DIF was present. Some few studies have previously evaluated the measurement invariance of the HADS in relation to sex with diverging results. The HADS was shown to be a valid tool for comparisons between sexes in a population of cardiac patients [36] and in a population of outpatients attending a musculoskeletal rehabilitation program [37]. Yet, when the HADS was evaluated in a population of patients who had undergone heart surgery, DIF for sex was found [38]. Further, DIF for age and sex was found for those 55 years and over in a primary care setting [39]. Even if our study showed absence of DIF for sex in an older general population there are further needs for evaluating DIF in other groups, such as age and ethnicity.

### Methodological considerations

The large random sample of a general population is a strength of the present study, though one consequence of a large statistic power is the increased risk to detect statistically significant results of minor importance. We have therefore combined the use of *p*-values with other statistical methods to evaluate the psychometric properties, for example graphs and effect size measures. One potential limitation is that the upper age was limited to 80 years. No strong conclusions about the HADS can therefore be drawn about the oldest. Our findings need therefore to be confirmed in age groups above 80 years. The dropout rate of 33% is in line with what could be expected in this type of surveys [40]. In fact, the population in this study was in the age group of 65–80 years, where disability and poor health is more common than among younger people. Therefore, the dropout rate can be considered as low. A large drop out may have serious consequences for external validity. However, in psychometric studies large drop outs are seldom a problem as long as it will not affect the variation in data, for example that not all response categories are used. According to the score distribution this was not a problem in the present study. Another strength of this study is that we have used appropriate statistical methods for ordinal level data, which strengthens the statistical validity.

## Conclusions

This study showed that the hypothesized two-factor structure, measuring anxiety and symptoms of depression respectively, is also adequate for a general older population. In addition, internal consistency was satisfactory and no DIF for sex was detected. Problems with floor effects were shown for all items. Even if the floor effects did not have any serious impact on the factor structure in the present study, users should be aware of this problem as it may have negative consequences for both sensitivity and responsiveness. Despite this, HADS can be recommended to assess psychological distress among a general population 65–80 years old.

## Abbreviations

CFA: Confirmatory factor analysis
CFI: Comparative Fit Index
DIF: Differential Item Functioning
HAD: Hospital Anxiety and Depression Scale
MCAR: Missing Completely at Random
RMSEA: Root Mean Square Error of Approximation
TLI: Tucker Lewis Index
WLSMV: Weight Least Square Means and Variations
